# The impact of methamphetamine/amphetamine use on receipt and outcomes of medications for opioid use disorder: a systematic review

**DOI:** 10.1186/s13722-021-00266-2

**Published:** 2021-10-11

**Authors:** Madeline C. Frost, Hannah Lampert, Judith I. Tsui, Matthew D. Iles-Shih, Emily C. Williams

**Affiliations:** 1 Department of Health Systems and Population Health, University of Washington School of Public Health, 1959 NE Pacific St, WA 98195 Seattle, United States; 2 Health Services Research & Development (HSR&D) Center of Innovation for Veteran-Centered and Value-Driven Care, Veterans Affairs (VA) Puget Sound Health Care System, 1660 South Columbian Way, Seattle, WA 98108 United States; 3 Department of Medicine, University of Washington School of Medicine, 1959 NE Pacific St, Seattle, WA 98195 United States; 4 Department of Psychiatry and Behavioral Sciences, University of Washington School of Medicine, 1959 NE Pacific St, Seattle, WA 98195 United States

**Keywords:** Methamphetamine, Amphetamine, Opioid use disorder, Opioid agonist, Buprenorphine, Methadone, Naltrexone, Polysubstance use

## Abstract

**Background:**

Methamphetamine/amphetamine use has sharply increased among people with opioid use disorder (OUD). It is therefore important to understand whether and how use of these substances may impact receipt of, and outcomes associated with, medications for OUD (MOUD). This systematic review identified studies that examined associations between methamphetamine/amphetamine use or use disorder and 3 classes of outcomes: (1) receipt of MOUD, (2) retention in MOUD, and (3) opioid abstinence during MOUD.

**Methods:**

We searched 3 databases (PubMed/MEDLINE, PsycINFO, CINAHL Complete) from 1/1/2000 to 7/28/2020 using key words and subject headings, and hand-searched reference lists of included articles. English-language studies of people with documented OUD/opioid use that reported a quantitative association between methamphetamine/amphetamine use or use disorder and an outcome of interest were included. Study data were extracted using a standardized template, and risk of bias was assessed for each study. Screening, inclusion, data extraction and bias assessment were conducted independently by 2 authors. Study characteristics and findings were summarized for each class of outcomes.

**Results:**

Thirty-nine studies met inclusion criteria. Studies generally found that methamphetamine/amphetamine use or use disorder was negatively associated with receiving methadone and buprenorphine; 2 studies suggested positive associations with receiving naltrexone. Studies generally found negative associations with retention; most studies finding no association had small samples, and these studies tended to examine shorter retention timeframes and describe provision of adjunctive services to address substance use. Studies generally found negative associations with opioid abstinence during treatment among patients receiving methadone or sustained-release naltrexone implants, though observed associations may have been confounded by other polysubstance use. Most studies examining opioid abstinence during other types of MOUD treatment had small samples.

**Conclusions:**

Overall, existing research suggests people who use methamphetamine/amphetamines may have lower receipt of MOUD, retention in MOUD, and opioid abstinence during MOUD. Future research should examine how specific policies and treatment models impact MOUD outcomes for these patients, and seek to understand the perspectives of MOUD providers and people who use both opioids and methamphetamine/amphetamines. Efforts to improve MOUD care and overdose prevention strategies are needed for this population.

## Introduction

Over 1.6 million people in the United States have opioid use disorder (OUD) [1]. Almost 50,000 people in the United States died of opioid overdose in 2019 [2], and overdose death has markedly increased during the COVID-19 pandemic [3–7]. Worldwide, OUD is one of the most prevalent drug use disorders and a notable source of global mortality and morbidity [8]. There are 3 US Food and Drug Administration (FDA)-approved treatment medications for OUD (MOUD), including methadone, buprenorphine and naltrexone [9]. Opioid agonist medications (methadone and buprenorphine) reduce risk of opioid overdose [10–12], and overdose risk has been observed to increase when patients exit agonist treatment demonstrating the importance of retention in treatment [10, 12]. MOUD are considerably underused, and increasing access to and retention in MOUD treatment, particularly opioid agonist medications, is essential to addressing the opioid crisis and preventing overdose death [9]. In light of this goal, MOUD are increasingly being provided outside of specialty substance use treatment settings including in primary care [13, 14] and community settings such as syringe services programs (SSPs) [15].

Multiple sources of data suggest that methamphetamine use is increasing among people with OUD. In the United States, a sharp increase in reported methamphetamine use has been documented among people entering OUD treatment—a nationwide survey found an 85% increase in prevalence between 2011 and 2018 [16, 17], and an analysis of the national Treatment Episode Data Set found a 490% increase in prevalence from 2008 to 2017 [18]. An analysis of National Survey on Drug Use and Health data found that prevalence of recent illicit methamphetamine use more than tripled among people with recent heroin use or heroin use disorder from 2015 to 2017, and more than doubled among people with prescription OUD during the same period [19]. Amphetamine use is also growing globally—the United Nations reports that amphetamine seizures quadrupled worldwide from 2009 to 2018, and that methamphetamine/amphetamine use has increased across multiple regions [20]. Methamphetamine in particular is known to be highly addictive, and its use is often associated with multiple health and social problems [21].

Given the striking increase in methamphetamine/amphetamine use both generally and among people with OUD specifically, as well as the highly addictive nature of methamphetamine and associated adverse effects, it is important to understand how use of these substances impacts receipt of and outcomes associated with MOUD. Blondino and colleagues published a systematic review of studies conducted in the United States and published before 11/28/2018 that examined associations between co-occurring substance use and retention in MOUD and opioid abstinence during MOUD, and summarized 7 articles assessing associations between amphetamine use and these 2 outcomes [22]. In order to more fully understand existing research and gaps in knowledge regarding the impact of methamphetamine/amphetamine use on MOUD—including its impact on the entire MOUD care continuum, potential trends reflecting changes in drug use patterns and MOUD provision, and potential variation across settings—an expanded review is needed that includes studies examining receipt of MOUD, studies published more recently, and studies conducted outside of the United States.

The objective of this systematic review was to identify studies that examine and report associations between methamphetamine/amphetamine use or use disorder and 3 classes of outcomes: (1) receipt of MOUD, (2) retention in MOUD, and (3) opioid abstinence during MOUD. We describe study characteristics and findings, as well as potential implications and key gaps in existing research.

## Methods

This review follows reporting guidelines specified in the Preferred Reporting Items for Systematic Reviews and Meta-Analysis (PRISMA) statement [23].

### Data sources and search strategy

Three databases (PubMed/MEDLINE, PsycINFO, CINAHL Complete) were searched from 1/1/2000 to 7/28/2020. The database search strategy was developed in consultation with the Health Sciences library at the University of Washington. Boolean search queries were created using a combination of keywords and subject headings (complete search queries are included in Appendix 1). Reference lists of included studies were later hand-searched to identify additional studies meeting inclusion criteria.

### Inclusion and exclusion criteria

Included studies met the following criteria: (1) the study sample was composed of people who use opioids and/or have documented OUD; (2) the study examined and reported on a quantitative association between methamphetamine/amphetamine use or use disorder and one of 3 types of MOUD outcomes of interest, with MOUD including methadone, buprenorphine and/or naltrexone; and (3) the study was published in English. We did not exclude studies if they did not limit their sample to people with diagnosed OUD, as many studies examining MOUD receipt do not assess OUD but examine samples likely to include many people who meet diagnostic criteria for OUD (e.g., people who inject heroin). MOUD outcomes of interest included (1) receipt of MOUD, which included initiation (i.e., newly starting MOUD during the study period) or any receipt (i.e., documentation of MOUD receipt during a specified period, which may or may not represent a new initiation); (2) retention in MOUD, which included both continuous measures of time in treatment (i.e., time from initiation until discontinuation) and categorical measures of time in treatment (i.e., remaining in treatment for a specified length of time); and (3) opioid abstinence during MOUD, measured through urine screens and/or self-report of opioid use. Studies were excluded if their sample was not restricted to people who use opioids and/or have documented OUD, if they examined any stimulant use (including cocaine and/or amphetamines) but did not separately examine the association of methamphetamine/amphetamine use with the outcome(s) of interest, and if they examined use of MOUD that was not prescribed. Studies were not excluded based on design (provided they included a quantitative analysis of the association of interest), geographic location, or clinical setting.

### Study screening and selection

Abstracts were independently screened by 2 authors (MCF and HL) and excluded if they clearly did not meet inclusion criteria; disagreements were resolved through consensus between the 2 authors. Remaining full-text articles were independently reviewed for final inclusion/exclusion by the same 2 authors, and disagreements were resolved through consensus between the 2 authors or through consultation with the senior author (ECW) as needed. Reference lists of included articles were hand-searched by one author (MCF) to identify additional studies possibly meeting inclusion criteria, and inclusion or exclusion of these articles was independently confirmed by a second author (HL).

### Data extraction and quality assessment

The same 2 authors independently extracted study data using a template developed by the study team to capture desired information; disagreements were resolved through consensus between the 2 authors. Extracted data included study design, dates, setting, population, adjunctive services to address substance use (i.e., psychosocial treatments or support groups, if the paper clearly described that these were provided or offered to study participants), average MOUD dose (if described), total number of subjects and number with methamphetamine/amphetamine use or use disorder, measure definitions, statistical analyses, covariates, and estimated association(s).

Risk of bias was assessed for each study using the Quality in Prognosis Studies tool [24], which assesses level of bias (low, moderate or high) in 6 domains: (1) participation, (2) attrition, (3) prognostic factor (i.e., methamphetamine/amphetamine use or use disorder) measurement; (4) outcome measurement, (5) confounding, (6) analysis and reporting. The level of bias for each domain was determined with respect to the specific association of interest for the present review—for example, if a study presented an unadjusted association for methamphetamine/amphetamine use and the outcome but did not include it in the multivariable model, the study was determined to have a high level of bias for confounding. The attrition domain was considered not applicable for cross-sectional studies and for longitudinal studies in which treatment retention/discontinuation was the only outcome of interest examined. Two authors (MCF and HL) independently conducted the risk of bias assessment; disagreements were resolved through consensus or through consultation with the senior author (ECW) as needed. Study screening, data extraction and quality assessment were performed using Covidence systematic review software [25].

## Results

### Description of included studies

**Fig. 1:**
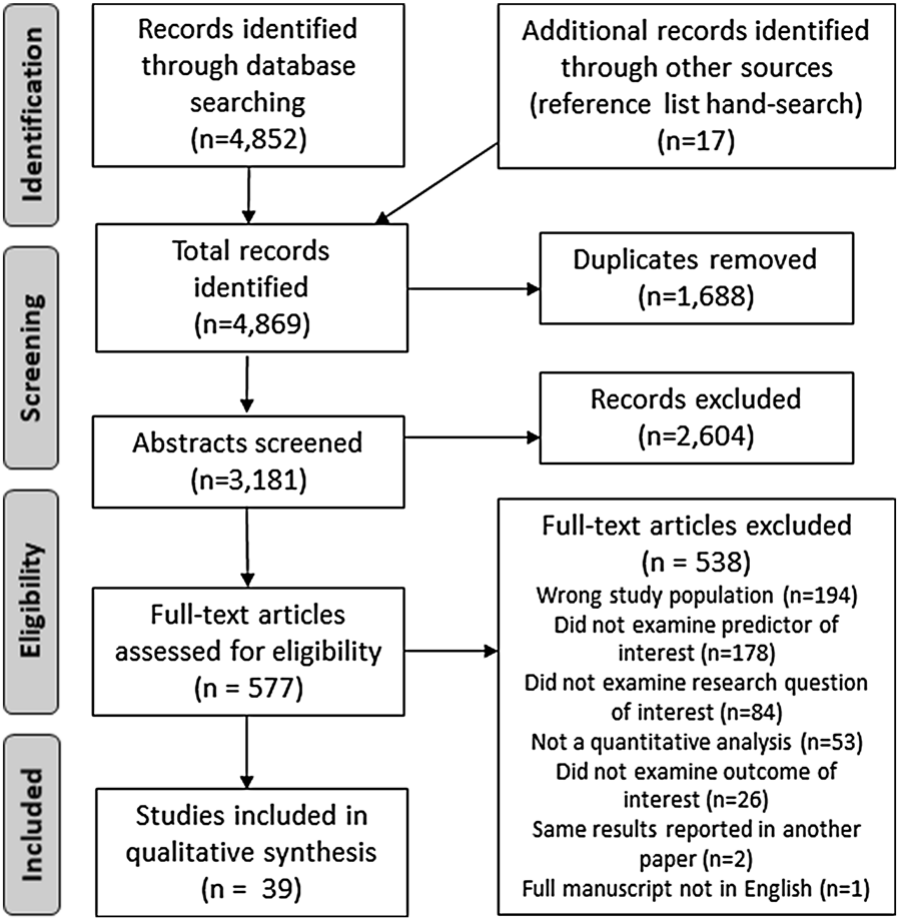
PRISMA flow diagram depicting study identification and selection process

The database search returned 4852 records, and 1688 duplicates were removed. Seventeen additional articles were later identified through hand-searching reference lists of included articles. Three thousand one hundred eighty-one abstracts were screened, and 2604 were excluded. Five hundred seventy-seven full-text articles were reviewed and 538 were excluded, resulting in a total of 39 articles included for qualitative synthesis (Fig. 1). The 2 independent reviewers had “substantial agreement” at both phases of study selection based on a kappa statistic (kappa  =  0.69 for abstract screening, kappa  =  0.77 for full-text review) [26].

### Receipt of MOUD treatment

Thirteen studies examined the association between methamphetamine/amphetamine use or use disorder and receipt of MOUD (Table 1). Eight used a cross-sectional study design and 5 used a longitudinal study design. Time periods for data collection ranged from 1992 to 2018, with only 2 studies having collected data within the past 5 years (2016 or later). Eight studies were conducted in the United States; other studies were conducted in Canada, Thailand, Vietnam, Norway, and France. Study populations and settings included patients with OUD in general healthcare settings (4 studies; 1 limited to patients with both OUD and post-traumatic stress disorder), patients presenting for specialty substance use treatment for opioid use (3 studies), parents who used opioids enrolled in a child welfare-based substance use intervention program (1 study), people with OUD recruited through a community survey (1 study), and people who inject drugs (PWID) reporting opioid use recruited through SSPs or community surveys (4 studies; 1 limited to PWID with HIV). Four studies examined amphetamine use disorder, 4 examined amphetamine use, and 6 examined methamphetamine use (1 study separately examined both amphetamine and methamphetamine use). Amphetamine use disorder was measured using diagnostic codes for abuse or dependence, methamphetamine/amphetamine use was primarily measured by self-report of use during varying timeframes ranging from the past week to the past 6 months. Three studies examined receipt of any MOUD, 2 examined any agonist (methadone or buprenorphine), 1 examined buprenorphine or naltrexone, 6 examined methadone alone, 3 examined buprenorphine alone, and 1 examined naltrexone alone. Five studies adjusted for other substance use or use disorders.

**Table 1:** Details from included studies examining receipt of MOUD

Author/year	Study design	Time period	Setting	Population	Total N	Meth/amph measure	MOUD measure	Analysis^a^	Covariates^b^	Association^c^	Direction (receipt)
Deck 2004	Cross-sectional	1992–2000	Oregon and Washington, US; publicly funded SUD tx programs	Medicaid-eligible adults presenting for opioid use tx (first tx episode)	Oregon: 7804; Washington: 9292	Amph use in past 30 days at intake (self-report)	Placement in methadone tx vs. other modality (regular outpatient, residential, or residential detox)	Logistic regression	Demographics, social factors, other substance use, mental health, prior tx, referral source, distance from clinic	Oregon: aOR = 0.53, p < 0.01	Negative
						Ns not presented for first episode only; 12% (Ore.), 11% (Wash.) of all tx episodes				Washington: aOR = 0.52, p < 0.01	
Fairbairn 2012	Cross-sectional	2009	Bangkok, Thailand; community survey	PWID with past 6-month injection drug use who use opioids	273	Meth use in past 6 months (self-report)	Reporting methadone receipt at least once in past 6 months	Logistic regression	Demographics, other substance use	aOR = 0.49 (95% CI 0.29–0.85), p = 0.010	Negative
						n = 137					
Gjersing 2013	Cross-sectional	2002–2011	Oslo, Norway; SSP	SSP participants with past 4-week injection drug use who use heroin	1760	“Daily/almost daily” amph use in past 4 weeks (self-report)	Reporting current receipt of methadone or bup	Logistic regression	None	OR = 0.7 (95% CI 0.6–1.0), p ≤ 0.05	Negative
						n = 567					
Jones 2020	Cross-sectional	2017	US; federally funded SUD tx (TEDS data)	Admissions for patients age 12 + with heroin as primary substance	533,394	Meth use listed as secondary or tertiary substance at admission	Any MOUD (bup, methadone, and/or naltrex) is part of tx plan at admission	Logistic regression (amph is dependent variable)	Demographics, social factors, referral source, injection, age of first heroin use	aOR = 0.65 (95% CI 0.50–0.84)	Negative
						n = 65,922					
Michel 2017	Longitudinal	2014–2015	Haiphong, Vietnam; community survey	Adult PWID who use heroin not currently receiving methadone	194	Meth use at baseline survey (UDS or self-report)	NOT reporting having started methadone at week 52 f/u	Logistic regression	Frequency of drug injection	aOR = 3.34 (95% CI 1.92–5.79)	Negative
						n = 76					
Rhee 2019	Cross-sectional	2006–2015	US; outpatient physician visits (NAMCS data)	Visits for adult patients with OUD (weighted)	2,055,381	Amph UD (dx codes, abuse/dependence)	Bup rx at visit	*X*^2^ test	None	0.1% of bup vs. 1.4% of no bup visits had amph UD; p = 0.010	Negative
						n = 10,277					
Shiner 2017	Longitudinal	2003–2013	US; national VA	Adult VA patients with OUD and new PTSD treatment episode	19,998	Amph UD (dx codes, abuse/dependence)	Any MOUD received in year after index visit (rx for bup/naltrex, clinic visit for methadone)	Logistic regression	Demographics, social factors, military experiences, physical/mental health, other SUDs, utilization	aOR = 0.77 (95% CI 0.68, 0.88)	Negative
						n = 1524					
Daniulaityte 2020	Cross-sectional	2017–2018	Dayton, Ohio, US; community survey	Adults with OUD and past-6 month use of non-rx buprenorphine	356	Meth use in past 6 months (self-report) n = 198	Reporting lifetime receipt of injectable naltrex, bup, or methadone (examined separately)	Logistic regression (meth is dependent variable)	Demographics, social factors, mental health, other substance use, receipt of other 2 MOUD types	Naltrex aOR = 2.89 (95% CI 1.45–5.75), p = 0.003	Positive (naltrex only)
										Bup aOR = 0.85 (95% CI 0.49–1.48), p = 0.57	
										Methadone aOR = 0.63 (95% CI 0.37–1.11) p = 0.11	
Morgan 2018	Longitudinal	2010–2014	US; national insurance claims data	Commercially insured individuals with OUD	340,017	Amph UD during study period (dx codes, abuse/dependence)	Filled rx for bup or naltrex (oral or injectable) during study period	Logistic regression	Demographics, health plan type, other SUDs	aOR = 1.183 (95% CI 1.127–1.241), p < 0.001	Positive
						n = 13,508					
Hall 2016	Longitudinal	2007–2015	Kentucky, US; child welfare-based SUD program	Adults using opioids in child abuse/neglect cases with ≥ 1 child age ≤ 5 years old in their household	596	“Current” amph/meth use (self-report; examined separately)	≥ 1 month of any MOUD (bup, methadone, or naltrex) while involved in program	*X*^2^ test	None	9.1% of ≥ 1 month MOUD vs. 8.3% of < 1 month had amph use; p = 0.84	Non-significant
						Amph n = 50				9.1% of ≥ 1 month vs. 6.8% of < 1 month had meth use; p = 0.53	
						Meth n = 42					
Pettes 2010	Longitudinal	2005–2008	Vancouver, Canada; community survey	Adult HIV-positive PWID with heroin use	353	“Frequent” meth use in past 6 months (self-report; repeated measure)	Reporting current enrollment in methadone tx (repeated measure)	GEE model with logit link	None	OR = 0.60 (95% CI 0.29–1.24), p = 0.166	Non-significant
						n = 12 at baseline					
Manhapra 2020	Cross-sectional	2011–2012	US; national VA	VA patients with OUD with no bup or methadone in first 60 days of FY 2012	94,145	Amph UD during FY 2012 (dx codes, abuse/dependence)	Receipt of bup (filled rx) or methadone (clinic visit) during FY 2012 (examined separately)	Calculated RRs (ref group: no bup or methadone)	None	Bup RR = 1.23 Methadone RR = 1.09	No statistical test reported
						n = 4887					
Thirion 2001	Cross-sectional	1995–1997	France; national survey of SUD tx centers	Patients with opioid use in SUD tx centers	1506	Amph use in past week (self-report)	Reporting receipt of bup or methadone compared to reporting heroin use with no bup or methadone	Compared % with amph use across groups	None	1% in bup group, 1% in methadone group, 6% in heroin use with no bup ormethadone group had amph use	No statistical test reported
						n = 47					

*amph* amphetamine; *aOR* adjusted odds ratio; *bup* buprenorphine; *CI* confidence interval; *dx* diagnostic; *f*/*u* follow-up; *FY* fiscal year; *GEE* generalized estimating equation; *naltrex* naltrexone; *NAMCS* National Ambulatory Medical Care Survey; *meth* methamphetamine; *MOUD* medications for opioid use disorder; *OR* odds ratio; *OUD* opioid use disorder; *PTSD* post-traumatic stress disorder; *PWID* people who inject drugs; *ref* reference; *RR* risk ratio; *rx* prescription; *SSP* syringes services program; *SUD* substance use disorder; *TEDS* Treatment Episode Data Set; *tx* treatment; *UD* use disorder; *UDS* urine drug screen; *US* United States; *VA* Veterans Health Administration

^a^Meth/amph use/use disorder measure is independent variable and MOUD measure is dependent variable unless otherwise noted

^b^A detailed description of covariates is provided in Appendix 2: Table 5

^c^Crude measures of association are only presented alongside adjusted measures if there was a difference in statistical significance; p-values and/or 95% CIs are presented when they were reported

Seven studies found a significant negative association between amphetamine use disorder or amphetamine/methamphetamine use and receipt of MOUD [18, 27–32]. Outcomes examined in these studies included receipt of any MOUD, any agonist, methadone alone, and buprenorphine alone. Two studies found a significant positive association; one between past 6 month methamphetamine use and lifetime receipt of injectable naltrexone among adults with OUD recruited through a community survey in a United States city [33], and the other between amphetamine use disorder and receipt of either buprenorphine or naltrexone (injectable or oral, measured through outpatient pharmacy claims) among commercially-insured adults with OUD in the United States [34]. Two studies found no significant association; one separately examined amphetamine and methamphetamine use and receipt of any MOUD within a child welfare-based substance use intervention program in Kentucky, United States [35], and the other examined “frequent” methamphetamine use and reporting current enrollment in methadone treatment among PWID with HIV recruited through a community survey in Vancouver, Canada, in which only 12 participants reported frequent methamphetamine use [36]. Two studies did not report tests of statistical significance [37, 38]. There were no clear patterns in findings across studies with respect to study design, time period, geographic location, population/setting, predictor measurement or covariate adjustment including adjustment for other substance use/use disorders.

### Retention in MOUD treatment

Twenty-one studies examined the association between methamphetamine/amphetamine use or use disorder and retention in MOUD (Table 2). All studies used a longitudinal design; one was a secondary analysis of data collected for a randomized controlled trial. Time periods for data collection ranged from 1993 to 2018, with only 3 studies having collected data within the past 5 years (2016 or later). Thirteen studies were conducted in the United States, 2 in both Israel and the United States, 2 in Canada, and other studies were conducted in Israel, China, Norway and Ireland. All studies included patients receiving MOUD; study settings included methadone treatment programs (8 studies), buprenorphine treatment programs (5 studies), specialty opioid treatment programs providing both methadone and buprenorphine (3 studies; 1 youth treatment program), buprenorphine or naltrexone receipt assessed through medical records or insurance claims (3 studies), and community surveys of people who use opioids self-reporting methadone receipt (2 studies). Four studies examined amphetamine use disorder, 1 examined methamphetamine use disorder, 11 examined amphetamine use, and 5 examined methamphetamine use. Methamphetamine/amphetamine use disorder was measured using diagnostic codes or diagnostic criteria; methamphetamine/amphetamine use was measured either through urine drug screen (UDS) or self-report of use during varying timeframes either prior to intake or during treatment. Definitions of retention outcomes varied; some studies measured retention as a time-to-event variable, while others used binary or categorical measures of retention until various times ranging from 30 days to 3 years. Seven studies adjusted for other substance use.

**Table 2:** Details from included studies examining retention in MOUD

Author/year	Study design	Time period	Setting	Population	Adjunctive services^a^	Total N	Meth/amph measure	MOUD measure	Analysis	Covariates^b^	Association^c^	Direction (retention)
Banta-Green 2009	Long-itudinal	2004–2005	Washington state, US; methadone tx programs	Adult patients initiating methadone	–	2308	Meth use “at time of intake” (self-report)	Retention at 1 year after initiation	Logistic regression	Demographics, social factors, physical health, other substance use, tx agency	aOR: 0.62 (95% CI 0.44–0.89), p = 0.009	Negative
							n = 164					
Deck 2005	Long-itudinal	1994–1999	Oregon and Washington, US; publicly funded methadone tx programs	Medicaid eligible adults initiating methadone	–	OR: 3185	Amph use in past 30 days at initiation (self-report)	Retention at 1 year after initiation	Logistic regression	Demographics, social factors, other substance use, mental health, prior tx, referral source, distance from clinic, tx agency	Oregon: aOR = 0.76 (95% CI 0.57–0.83)	Negative (Oregon only)
						WA: 5103	Ns not presented for first episode only; 8% (Ore.), 5% (Wash.) of all tx episodes				Washington: aOR = 1.01 (95% 0.70–1.45)	
Hser 2014	Long-itudinal (RCT data)	2006–2009	US; federally-licensed OUD tx programs (various locations)	Adult patients initiating bup or methadone in RCT	–	Total: 1267	Amph use during 24-week f/u period (UDS)	Time to discontinuation	Cox PH model	Demographics, physical/mental health, other substance use, site, dose, MOUD type (in total sample)	Total sample: aHR = 4.87 (95% CI: 3.75–6.34) Bup: aHR = 4.50 (95% CI 3.32–6.10) Methadone: aHR = 6.85 (95% CI 4.00–11.72),	Negative
						Bup: 738	Ns at initiation					
							Total sample n = 114					
						Meth-adone: 529	Bup n = 64				(all p < 0.01)	
							Methadone n = 50					
Liu 2017	Long-itudinal	2013–2014	Guangzhou, China; methadone tx programs	Adult patients receiving methadone, with heroin use prior to tx	–	401	Meth use in past 6 months at baseline survey (self-report)	Time to discontinuation	Cox PH model	Marital status, # of times in “compulsory drug detoxification”	aHR = 2.26 (95% CI 1.15–4.43), p = 0.017	Negative
							n = 31					
Lo 2018	Long-itudinal	2005–2015	Vancouver, Canada; community survey	Adult PWUD reporting methadone tx in past 6 months	–	1301	At least daily meth use in past 6 months (self-report; repeated measure)	Reporting discontinuation in past 6 months (repeated measure)	GEE model with logit link	Demographics, social factors, other substance use, HIV, dose, % of visits on methadone	Crude OR = 1.75 (95% CI 1.07–2.85), p = 0.025 aOR = 1.02 (95% CI 0.61–1.69), p = 0.951	Negative (unadjusted only)
							n = 66 at baseline					
Morgan 2018	Long-itudinal	2010–2014	US; national insurance claims data	Commercially insured individuals with OUD initiating bup or naltrex	–	38,190	Amph UD during study period (dx codes; abuse or dependence)	Time to discontinuation	Cox PH model	Demographics, other substance use, ever had detox, provider type, clinic setting, type of insurance, MOUD type	aHR 1.07 (95% CI 1.03–1.12), p = 0.002	Negative
							n = 2353					
Peles 2008	Long-itudinal	Tel Aviv: 1993–2004 Las Vegas: 2000–2005	Tel Aviv, Israel and Las Vegas, Nevada, US; methadone tx programs	Adult patients initiating methadone	Individual therapy (both clinics): group therapy (required Tel Aviv; encouraged Las Vegas)	Tel Aviv: 492	Amph use at initiation (UDS)	Retention at 1 year after initiation^d^	Tel Aviv: Fisher’s exact test	Tel Aviv: none	Tel Aviv: Fisher’s exact p = 0.2	Negative (Las Vegas clinic only)
						Las Vegas: 302	Tel Aviv: n = 45		Las Vegas: logistic regression	Las Vegas: demographics, duration of opioid use, Hepatitis C	Las Vegas: aOR for NO amph use = 2.1 (95% CI 1.05–4.2)	
							Las Vegas: n = 47					
Skeie 2013	Long-itudinal	1998–2007	Hedmark county/Oppland county, Norway; public OUD tx program	Adult patients receiving bup or methadone	–	131	Years of amph dependence during lifetime (structured interview)	Interruption of tx (planned or unplanned) during study period	Independent samples t test	None	Mean 9.2 years in interrupter group; 5.5 years in other group; t test p = 0.048	Negative
							n not reported					
Tsui 2020	Long-itudinal	2015–2018	Washington state, US; primary care bup tx program in urban hospital; rural telemedicine bup tx program	Adult patients initiating bup	–	768	Meth use in past 30 days at initiation (self-report; any vs. none and # of days)	Time to discontinuation	Cox PH models (ref group: no meth use)	Demographics, clinic site, time period of enrollment	Any use aHR: 2.39 (95% CI 1.94–2.93) 1–10 days aHR: 2.05 (95% CI 1.63–2.57) 11–20 days aHR: 3.04 (95% CI 2.12–4.23) 21–30 days aHR: 3.61 (95% CI 2.40–5.23)	Negative
							n = 237 (any use)					
Schiff 2007	Long-itudinal	2004–2005	Israel; methadone tx programs (various locations)	Adult patients receiving methadone	–	2664	Amph use during tx (UDS)	100% retention during 13-month study period	Logistic regression	Demographics, other substance use	aOR = 1.48 (95% CI 1.08-2.02), p = 0.015	Positive
							(n not reported)					
Hui 2017	Long-itudinal	2002–2014	Boston, MA, US; primary care bup tx in large safety-net medical center	Adult patients initiating bup	Behavioral health counseling required	1127	Amph use in first month of tx (UDS)	Discontinuation within 30 days after initiation	*X*^2^ test	None	4.8% of those who left vs. 3.2% who did not had amph use*;* p = 0.48	Non-significant
							n = 37					
Kumar 2016	Long-itudinal	2012–2015	Little Rock, AR, US; outpatient bup tx program in university medical center SUD tx clinic	Adult patients initiating bup	Relapse prevention groups and individual CBT required	113	Amph use at initiation (UDS)	Discontinuation within 90 days after initiation	Logistic regression	Demographics, other substance use, pain, physical or emotional neglect (2 models)	Adj. for physical neglect: aOR = 5.37 (95% CI 0.60–48.34); adj. for emotional neglect: aOR = 4.53 (95% CI 0.50–41.01)	Non-significant
							n = 13					
Logan 2019	Long-itudinal	2016–2018	Hawaii Island, Hawaii, US; rural FQHC primary care bup tx program	Patients initiating bup	Individual therapy and mutual support groups required	54	Meth UD assessed at initiation (dx criteria)	Retention at 3 months after initiation	*X*^2^ test	None	No significant association (test statistic/p value not reported)	Non-significant
							n = 24					
Schuman-Olivier 2014	Long-itudinal	2007–2010	Boston, MA, US; outpatient bup tx program in academic community healthcare system	Adult patients initiating bup	Psycho-social tx (group or individual), relapse prevention group required	294	Amph use at initiation (UDS)	Retention at 3 months and 12 months after initiation	Logistic regression	None	3-month: OR = 1.31 (95% CI 0.42–4.09), p = 0.647	Non-significant
							n = 18				12-month: OR = 2.10 (95% CI 0.80–5.49), p = 0.130	
Peles 2015	Long-itudinal	Tel Aviv: 1993–2013	Tel Aviv, Israel and Las Vegas, Nevada, US; methadone tx programs	Adult patients initiating methadone	Individual therapy (both clinics): group therapy (required Tel Aviv; encouraged Las Vegas)	Tel Aviv: 306	Amph use during tx (UDS)	Retention at 6 months after initiation	Fisher’s exact test	None	No significant association (test statistic/p value not reported)	Non-significant
		Las Vegas: 2000–2014				Las Vegas: 190	Tel Aviv n = 6					
							Las Vegas n = 12					
Pettes 2010	Long-itudinal	2005–2008	Vancouver, Canada; community survey	Adult PWID with HIV reporting methadone tx	–	248	“Frequent” meth use in past 6 months (self-report; repeated measure)	Time to discontinuation (reported having discontinued during past 6 months)	Cox PH model	None	No significant association (measure/p value not reported)	Non-significant
							n = 12 at baseline					
Proctor 2015	Long-itudinal	2009–2011	US; methadone tx programs operated by a large health care provider (various locations)	Adult patients initiating methadone	–	1644	Amph use at initiation and 6 month f/u (UDS)	Discontinuation before 6 months and before 1 year after initiation	Logistic regression	Demographics, social factors, dose	6-months: aOR = 1.57 (95% CI 0.92–2.69) for intake amph	Non-significant
							n = 178 at initiation; 41 at 6 months				12-months: aOR = 1.61 (95% CI 0.50–5.24) for intake amph; aOR = 2.91 (0.84–10.12) for 6-month amph	
Smyth 2018	Long-itudinal	2000–2016	Dublin, Ireland; youth OUD tx program	Adolescent patients (< 18.5 years) initiating methadone or bup	Tx involved counseling, family therapy (in some cases)	120	Amph use in past month at initiation (self-report)	Retention until month 12 after initiation	Fisher’s exact test; Calculated crude OR	None	Fisher’s exact test p = 0.45; OR = 1.9 (95% CI 0.5–7.7)	Non-significant
							n = 9					
Manhapra 2017	Long-itudinal	2011–2015	US; national VA	VA patients with OUD who initiated bup in FY 2012	–	3151	Amph UD during FY 2012 (dx codes, abuse/dependence)	Duration of tx (categorical) based on rx fills	Calculated RRs (ref group: 0–30 days)	None	31–365 days: RR = 1.51 1–3 years: RR = 1.31 > 3 years: RR = 1.10	No statistical test reported
							n = 199					
Manhapra 2018	Long-itudinal	2010–2014	US; national insurance claims data	Commercially-insured adults with OUD who initiated bup in FY 2011	–	16,190	Amph UD during FY 2011 (dx codes, abuse or dependence)	Duration of tx (categorical) based on rx fills	Calculated RRs (ref group: 0–30 days)	None	31–365 days: RR = 0.9 1–3 years: RR = 0.7 > 3 years: RR = 0.2	No statistical test reported
							n = 106					
White 2014	Long-itudinal	2011–2013	Washington, DC, US; private non-profit methadone tx program	Adult patients receiving methadone	Counseling group required; 12-step/mutual aid groups encouraged	604	Amph use during Aug 1-Nov 1, 2011 (UDS)	Discontinuation before Jan 2013	Compared %s across groups	None	3% of those who discontinued vs. 0.8% of those who did not had amph use	No statistical test reported
							n = 7					

*aHR* adjusted hazard ratio; *amph* amphetamine; *aOR* adjusted odds ratio; *bup* buprenorphine; *CBT* cognitive behavioral therapy; *CI* confidence interval; *dx* diagnostic; *FQHC* federally qualified health center; *f*/*u* follow-up; *FY* fiscal year; *GEE* generalized estimating equation; *K*–*M* Kaplan–Meier; *naltrex* naltrexone; *meth* methamphetamine; *MOUD* medications for opioid use disorder; *OR* odds ratio; *OUD* opioid use disorder; *PH* proportional hazards; *PWID* people who inject drugs; *PWUD* people who use drugs; *RCT* randomized controlled trial; *ref* reference; *RR* risk ratio; *rx* prescription; *SUD* substance use disorder; *tx* treatment; *UD* use disorder; *UDS* urine drug screen; *US* United States; *VA* Veterans Health Administration

^a^Included if paper clearly described that study participants received or were offered services

^b^A detailed description of covariates is provided in Appendix 2: Table 5

^c^Crude measures of association are only presented alongside adjusted measures if there was a difference in statistical significance; p values and/or 95% CIs are presented when they were reported

^d^Study also examined cumulative retention, however we were unable to interpret these results based on the description of these analyses

Nine studies found a significant negative association between methamphetamine/amphetamine use disorder or use and retention in MOUD [34, 39–46]. In one of these studies the association became non-significant after covariate adjustment [44], in 2 other studies the association was only significant in 1 of 2 populations that were examined (in both studies, the population with higher rates of amphetamine use had a significant negative association for amphetamine use and retention) [45, 46]. One study conducted among patients receiving methadone treatment in Israel during 2004–2005 found a positive association between amphetamine use during treatment measured by UDS and retention over 13 months [47]. Eight studies found no significant association [36, 48–54]. Three studies did not report tests of statistical significance [55–57], with one noting that there were “too few patients to perform statistical comparison” for this association [57].

There were no clear patterns in findings across studies with respect to time period, geographic location, population/setting, predictor measurement, type of MOUD, or covariate adjustment including adjustment for other substance use. While most studies finding a significant negative association measured retention as a time-to-event variable or retention at 1 year, studies reporting non-significant associations generally looked at retention over shorter time periods (i.e., 6 months or less). Studies reporting non-significant associations generally had low numbers of participants with the predictor of interest, and many had wide confidence intervals around estimated associations suggesting low statistical power. Additionally, most studies that described provision of some type of adjunctive services for substance use (e.g., psychosocial treatment, support groups) to study participants reported non-significant associations, though it is possible these services were provided but not described in other papers. However, one study reporting a non-significant association did not align with these patterns [50]. Average MOUD dose was not consistently reported across studies, preventing assessment of potential patterns in findings across average dose.

### Opioid abstinence during MOUD treatment

Eight studies examined the association between methamphetamine/amphetamine use or use disorder and opioid abstinence during MOUD (Table 3). Two used a cross-sectional study design and 6 used a longitudinal study design; 2 longitudinal studies were secondary analyses of data collected for randomized controlled trials. Time periods for data collection ranged from 2000 to 2016, with only 1 study having collected data within the past 5 years (2016 or later). Two studies were conducted in the United States, other studies were conducted in Taiwan, Vietnam, Norway, England, Ireland and Sweden. All studies included patients receiving MOUD; study settings included methadone treatment programs (3 studies), specialty opioid treatment programs providing both methadone and buprenorphine (2 studies; 1 youth treatment program), inpatient methadone treatment (1 study), an “interim” outpatient buprenorphine program (1 study) and people with OUD receiving sustained-release naltrexone implants as part of a clinical trial in inpatient treatment and prisons (1 study). One study examined amphetamine use disorder, 6 examined amphetamine use, and 1 examined methamphetamine use. Amphetamine use disorder was measured using diagnostic criteria, methamphetamine/amphetamine use was measured either through UDS or self-report of use during varying timeframes either prior to intake or during treatment. Opioid abstinence/use was measured as a binary variable, and definitions varied with respect to method of measurement (UDS or self-report) and timeframe (e.g., at any point vs. at specific time points during treatment). No studies adjusted for other substance use or use disorders.

**Table 3:** Details from included studies examining opioid abstinence during MOUD

Author/year	Study design	Time period	Setting	Population	Adjunctive services^a^	Total N	Meth/amph measure	MOUD measure	Analysis	Covariates^b^	Association^c^	Direction (abstinence)
Liu 2018	Cross-sectional	Not reported	Taiwan; methadone tx programs in hospitals (various locations)	Adult patients receiving methadone for ≥ 3 months	–	344	Amph use (UDS)	Opioid use (UDS)	*X*^2^ test	None	20% in opioid group had amph use vs. 9% in no opioid group; p = 0.005	Negative
							n = 51					
Hoang 2018	Longitudinal	2008–2013	Vietnam; methadone tx programs (national random sample)	Adult patients initiating methadone	–	500	Meth use prior to initiation (self-report)	Heroin use over 24-month f/u period (self-report and/or UDS)	Logistic regression	Family support, years used heroin, HIV status, antiretroviral therapy receipt	aOR: 2.68 (95% CI 1.08–6.65), p = 0.034	Negative
							n = 12					
Kunøe 2010	Longitudinal (RCT data)	2005–2007	Norway; inpatient SUD tx programs and prisons (various locations)	Adults with OUD receiving sustained-release naltrex implants in RCT	–	60	Frequency of amph use during 6-month study period and final month (self-report; none, 1–3 times/month, 1–3 times/week, daily/almost daily)	Opioid use during tx (self-report)	Rank-test procedure (Spearman’s R)	Demographics	Opioid use group had more frequent amph use in 6-month period (*R* = 0.29; p = 0.028) and the final study month (*R* = 0.35; p = 0.008)	Negative
							(n not reported)					
Proctor 2016	Longitudinal	2009–2011	US; inpatient SUD tx programs operated by large healthcare provider (various locations)	Adult patients receiving methadone with stay of ≥ 15 days	–	2410	Amph use at initiation, 3, 6, and 9 months (UDS)	Opioid use at 3, 6, 9, and 12-months (UDS)	Logistic regression	Demographics, dose	6-month amph use/9-month opioid use: aOR = 5.77 (95% CI 1.26–26.40), p = 0.024; p > 0.05 for all other time point combinations	Negative
							n = 219 at initiation (not reported for f/u timepoints)					
Potter 2013	Longitudinal (RCT data)	2006–2009	US; federally-licensed OUD tx programs (various locations)	Adults with OUD initiating bup or methadone in RCT	–	705	Amph UD assessed at initiation (dx criteria)	Opioid abstinence (no use in 30 days before end of tx; self-report)	*X*^2^ test	None	6.6% in opioid group had amph use vs. 8.0% in no opioid group; p > 0.05	Non-significant
							n = 52					
Senbanjo 2009	Cross-sectional	2003–2003	East Kent, England; methadone tx programs	Adults receiving methadone for ≥ 1 month	–	191	Amph use in past 14 days (self-report)	Heroin use in past 14 days (self-report)	*X*^2^ test; calculated unadjusted OR	None	*X*^2^ p = 0.399; OR = 2.07 (95% CI 0.37–11.6)	Non-significant
							n = 6					
Smyth 2018	Longitudinal	2000–2016	Dublin, Ireland; youth OUD tx program	Adolescent patients (< 18.5 years) initiating methadone or bup	Tx involved counseling, family therapy (in some cases)	39	Amph use in past month at initiation (self-report)	Heroin abstinence during 12th month of tx (UDS)	Fisher’s exact test; calculated unadjusted OR	None	Fisher’s exact p = 0.60; OR = 0.3 (95% CI 0.03–3.1)	Non-significant
							n = 4					
Abraham-sson 2016	Longitudinal	2011	Lund, Sweden; outpatient “interim” bup tx program	Adult patients with OUD initiating bup (on wait list for “full-scale” tx)	–	44	Days of amph use in past 30 days at initiation (self-report)	Opioid abstinence during entire study period (UDS)	Independent samples t test	None	Mean 0.6 days in opioid group; 1.2 days in no opioid group; t test p > 0.1	Non-significant
							n = 13 reported any amph use					

*amph* amphetamine; *aOR* adjusted odds ratio; *bup* buprenorphine; *CI* confidence interval; *dx* diagnostic; *f*/*u* follow-up; *meth* methamphetamine; *OUD* opioid use disorder; *RCT* randomized controlled trial; *SUD* substance use disorder; *tx* treatment; *UDS* urine drug screen; *US* United States

^a^Included if paper clearly described that study participants received or were offered services

^b^A detailed description of covariates is provided in Appendix 2: Table 5

^c^Crude measures of association are only presented alongside adjusted measures if there was a difference in statistical significance; p values and/or 95% CIs are presented when they were reported

Four studies found a significant negative association between amphetamine use disorder or methamphetamine/amphetamine use and opioid abstinence during MOUD treatment [58–61]. The other 4 studies found no significant association [54, 62–64]. There were no clear patterns in findings across studies with respect to study design, time period, geographic location, or definition of predictors/outcomes. Patients in studies finding a significant negative association were receiving methadone or sustained-release naltrexone implants, and patients in studies reporting non-significant associations were receiving methadone or buprenorphine. All but one of the studies finding a significant negative association adjusted for at least some covariates (though none adjusted for other substance use/use disorders), whereas all studies reporting non-significant associations presented unadjusted associations. Three of the 4 studies reporting non-significant associations had very low numbers of participants with the predictor of interest and wide confidence intervals, suggesting low statistical power. One study reporting a non-significant association that had a relatively higher number with the predictor of interest was the only study to examine diagnosed amphetamine use disorder as opposed to amphetamine/methamphetamine use during treatment [62]. Only one study described provision of any adjunctive services and average MOUD dose was not consistently reported across studies, preventing assessment of potential patterns in findings across these characteristics.

### Risk of bias

Results from the risk of bias assessment are presented in Table 4. Most studies were found to have low risk of bias for participation; some were found to have moderate risk due to incomplete descriptions of recruitment methods/participation rates or higher refusal rates. The attrition bias domain was considered not applicable to cross-sectional studies and studies examining only retention/discontinuation from treatment as an outcome; most remaining studies were found to have low risk of bias for attrition, and some were found to have moderate or high risk due to higher levels of attrition. Risk of bias for prognostic factor measurement (i.e., measurement of methamphetamine/amphetamine use or use disorder) was found to be low for most studies; some were found to have moderate risk due to incomplete measurement definition or use of documented diagnostic codes to assess substance use disorder, which may be under-diagnosed or documented inconsistently. Risk of bias for outcome measurement was also found to be low for most studies; some were found to have moderate risk due to incomplete measurement definition, the outcome not having a consistent timeframe across all study participants, or use of pharmacy claims/prescription fill data which may not capture all receipt of MOUD. Most studies were found to have moderate or high risk of bias for confounding due to lack of adjustment for some or all potential confounding factors. Many studies were found to have moderate risk of bias for statistical analysis and reporting due to lack of conceptually driven model-building, or lack of clarity in description of analyses and/or results.

**Table 4:** Risk of bias assessment summary ratings^a^

Author/pub year	Participation	Attrition^b^	Prognostic factor measurement	Outcome measurement	Confounding	Statistical analysis and reporting
Abrahamsson 2016	Moderate	Low	Low	Moderate	High	Moderate
Banta-Green 2009	Low	N/A	Low	Low	Low	Low
Daniulaityte 2020	Low	N/A	Low	Low	Low	Low
Deck 2004	Low	N/A	Low	Low	Low	Low
Deck 2005	Low	N/A	Low	Low	Low	Low
Fairbairn 2012	Moderate	N/A	Low	Low	Moderate	Moderate
Gjersing 2013	Low	N/A	Low	Low	High	Low
Hall 2016	Low	Low	Moderate	Low	High	Low
Hoang 2018	Low	Moderate	Moderate	Low	High	Moderate
Hser 2014	Low	N/A	Low	Low	Moderate	Low
Hui 2017	Low	N/A	Low	Low	High	Moderate
Jones 2020	Low	N/A	Low	Low	Low	Low
Kumar 2016	Low	N/A	Low	Low	Moderate	Moderate
Kunøe 2010	Low	Low	Low	Low	Moderate	Moderate
Liu 2017	Low	Low	Low	Low	High	Moderate
Liu 2018	Moderate	N/A	Low	Low	High	Low
Lo 2018	Low	N/A	Low	Low	Moderate	Moderate
Logan 2019	Low	N/A	Moderate	Low	High	Moderate
Manhapra 2017	Low	N/A	Moderate	Moderate	High	Moderate
Manhapra 2018	Low	N/A	Moderate	Moderate	High	Moderate
Manhapra 2020	Low	N/A	Moderate	Moderate	High	Moderate
Michel 2017	Moderate	Moderate	Moderate	Low	High	Moderate
Morgan 2018	Low	Low	Moderate	Moderate	Moderate	Low
Peles 2008	Low	N/A	Low	Low	High	Moderate
Peles 2015	Moderate	N/A	Low	Low	High	Moderate
Pettes 2010	Moderate	Low	Moderate	Low	High	Moderate
Potter 2013	Low	High	Low	Low	High	Moderate
Proctor 2015	Low	N/A	Low	Low	Moderate	Moderate
Proctor 2016	Low	Moderate	Low	Low	Moderate	Moderate
Rhee 2019	Low	N/A	Moderate	Low	High	Moderate
Schiff 2007	Low	N/A	Low	Moderate	Moderate	Moderate
Schuman-Olivier 2014	Moderate	N/A	Low	Low	High	Moderate
Senbanjo 2009	Moderate	N/A	Low	Low	High	Low
Shiner 2017	Low	Low	Moderate	Moderate	Low	Low
Skeie 2013	Moderate	N/A	Moderate	Low	High	Low
Smyth 2018	Low	High	Moderate	Low	High	Moderate
Thirion 2001	Low	N/A	Low	Moderate	High	Moderate
Tsui 2020	Low	N/A	Low	Low	Moderate	Low
White 2014	Low	N/A	Low	Low	High	Low

^a^Based on QUIPS risk of bias assessment instrument for prognostic factor studies; Hayden et al. [85]

^b^Attrition domain was considered not applicable for cross-sectional studies and studies only examining retention/discontinuation as outcome of interest

## Discussion

This systematic review identified studies from multiple countries examining the association between methamphetamine/amphetamine use or use disorder and a range of MOUD care continuum outcomes. Overall, existing research suggests that methamphetamine/amphetamine use and use disorder negatively impact receipt of MOUD, retention in MOUD and opioid abstinence during treatment. No clear pattern in findings was observed across time periods or geographic locations, though potential patterns emerged across outcomes, including MOUD type, longer vs. shorter-term retention, and the provision of adjunctive services during MOUD. These patterns should be directly examined in future research.

Studies examining receipt of MOUD generally found that amphetamine use disorder or methamphetamine/amphetamine use was negatively associated with receipt of opioid agonist medication. This finding appeared in studies spanning multiple time periods, geographic locations, clinical settings, and populations. It is possible that some observed associations are confounded by other substance use/use disorders, though 3 of the 7 studies finding a negative association adjusted for this. The 2 studies that found a positive association examined receipt of injectable naltrexone alone and naltrexone or buprenorphine [33, 34]. It is possible that an apparent association between methamphetamine/amphetamine use and receipt of naltrexone is confounded by the presence of alcohol use disorder for which naltrexone is an indicated treatment [65]. The study by Morgan and colleagues adjusted for alcohol use disorder diagnoses while the study by Daniulaityte et al. did not. Naltrexone has been studied as a potential pharmacotherapy for amphetamine use disorder [66, 67], however it is generally considered a second-line treatment for OUD [68], and may be less effective than agonist therapies in reducing risk of opioid overdose [11]. One study reporting a non-significant association likely had low power due to a very small number with the predictor of interest [36], and the other may have been the result of a unique study setting (a child welfare-based substance use intervention that aimed to facilitate linkage to MOUD) [35]. Overall, existing studies suggest that methamphetamine/amphetamine use may be a widespread barrier to receipt of opioid agonist medications among people with OUD, and further research is needed to determine whether receipt of naltrexone is more prevalent among people with OUD who use methamphetamine/amphetamines.

Studies examining retention in MOUD generally found negative associations between methamphetamine/amphetamine use disorder or use and retention across multiple study time periods, geographic locations, clinical settings and populations, as well as across different types of MOUD. Some observed associations may be confounded by other substance use, though 5 of the 9 studies finding a negative association adjusted for this. As we do not expect methamphetamine/amphetamine use to positively impact retention relative to no use, we considered potential differences among studies finding a negative association compared to studies finding no association between methamphetamine/amphetamine use and retention. Most studies reporting non-significant associations had relatively small numbers of participants with the predictor of interest, suggesting they may have been underpowered to detect associations. Besides the likely impact of low power, there were other potential differences among studies reporting negative associations compared to those reporting no association—most studies reporting no association examined retention over shorter periods of time than those that found negative associations, suggesting the possibility that methamphetamine/amphetamine use may have more of an impact on longer-term rather than shorter-term MOUD retention. Additionally, most studies reporting no association described some type of adjunctive services for substance use that were provided or offered to study participants, suggesting adjunctive services might improve retention for some people who use methamphetamine/amphetamines. However, provision of these services may not have been consistently reported across studies and low statistical power may be the primary factor driving non-significant results. Overall, existing studies suggest that methamphetamine/amphetamine use and use disorder negatively impacts MOUD retention.

Studies examining abstinence from opioid use during MOUD treatment generally found that methamphetamine/amphetamine use was negatively associated with opioid abstinence. However, as none of these studies adjusted for other substance use/use disorders, it is possible that observed associations are confounded by other substance use. Most studies finding significant negative associations were conducted in methadone clinics; one was a secondary analysis of randomized controlled trials testing sustained-release naltrexone implants [60]. Most studies reporting non-significant associations had very low numbers of participants with the predictor of interest and thus likely had low statistical power. One that may have had higher power was the only study to examine amphetamine use disorder [62], suggesting that only active use during treatment impacts opioid abstinence, however more research is needed to confirm this. Overall, existing studies suggest that methamphetamine/amphetamine use may negatively impact opioid abstinence during treatment among patients receiving methadone or sustained-release naltrexone implants, while the impact for patients receiving buprenorphine or other types of naltrexone is unclear. However, further research is needed adjusting for other substance use.

### Gaps in research and future directions

Research is needed to understand how varying characteristics of MOUD care influence the impact of methamphetamine/amphetamine use on MOUD outcomes. One study that did not meet inclusion criteria for this review (as it did not examine use of amphetamines/methamphetamines specifically) found that removing a buprenorphine program’s requirement that patients be abstinent from stimulants (cocaine or amphetamines) resulted in improved initiation, but decreased retention, for patients who used stimulants [69]. Future studies should similarly aim to understand the impact of specific clinical policies on MOUD receipt, retention, and treatment outcomes for people who use methamphetamine/amphetamines. Research is also needed to directly assess the impact of MOUD dose and receiving psychosocial treatments on MOUD retention and outcomes among people who use methamphetamine/amphetamines. Randomized controlled trials have found that providing contingency management and cognitive behavioral therapy to patients who used stimulants in MOUD reduced stimulant use, suggesting that offering concurrent, co-located treatments for multiple substance use disorders can benefit patients [70–72]. While there are currently no FDA-approved medications to treat amphetamine use disorder, ongoing work to advance pharmacologic treatment may also create opportunity for better simultaneous treatment [67]. However, treatment providers should recognize that requiring, rather than offering, additional treatment may create barriers to MOUD for some patients who use other substances, which could increase their risk of opioid overdose. Finally, most studies included in this review were conducted in specialty substance use treatment settings, though some were conducted in more general medical settings or involved community surveys. Studies are needed that examine outcomes for people who use methamphetamine/amphetamines in new settings where MOUD are increasingly being provided, such as emergency departments, prisons/jails, and community settings such as SSPs [15, 73, 74]. One study of SSP-based buprenorphine treatment found that stimulant use (cocaine or amphetamines) at enrollment was not associated with retention in bivariate analyses, suggesting MOUD outcomes for people who use methamphetamine/amphetamines might be improved in lower barrier settings [15].

Increased understanding of the perspectives of both MOUD providers and people who use drugs regarding co-occurring opioid and methamphetamine/amphetamine use is also needed. In surveys and qualitative studies buprenorphine providers have indicated they are less likely to prescribe for patients who use alcohol or benzodiazepines [75, 76], however providers’ thoughts on methamphetamine/amphetamine use are unclear. Some research suggests that people who use opioids/have OUD who also use methamphetamine/amphetamines are less likely to express interest in receiving help for substance use [77, 78]. Qualitative studies have found that people who use both opioids and methamphetamine describe a balancing effect of the drugs that increases functionality, which could be related to a lower perceived need for MOUD [17, 79]. Another qualitative study found that methadone patients who used stimulants described several benefits they experienced from their stimulant use, including balancing sedating effects of methadone [80]. Future research should seek to further understand how people who have OUD and use methamphetamine/amphetamine perceive their need for MOUD, whether they feel MOUD are accessible to and effective for them, and their recommendations to improve MOUD services.

Finally, evidence suggesting that methamphetamine/amphetamine use and use disorder is associated with reduced receipt of MOUD, reduced retention in MOUD, and opioid use during MOUD treatment highlights the necessity of maintaining and expanding evidence-based harm reduction strategies that prevent overdose death and reduce risk of other sequelae. Such strategies include widespread naloxone distribution, overdose prevention education, and supervised consumption facilities [81–84]. Harm reduction may play an increasingly important role in preventing overdose death if methamphetamine/amphetamine use continues to increase among people who use opioids, and efforts should be made to ensure that these services reach people who use multiple substances.

### Limitations

While our search strategy identified a large number of studies for screening, it may have missed studies not included in searched databases. We addressed this limitation by performing a hand search of the reference lists of included articles. Additionally, the inclusion criterion that studies be published in English may have resulted in the exclusion of some relevant studies. Most included studies analyzed data collected prior to 2016, and patterns may be changing as methamphetamine use continues to increase among people who use opioids and MOUD delivery continues to evolve. Many included studies did not examine methamphetamine/amphetamine use or use disorder as a primary variable of interest, but rather as one of several variables of interest, and therefore many did not adjust for covariates based on hypothesized confounding specific to methamphetamine/amphetamine use or use disorder. As described above, several studies appeared underpowered to detect the association of interest based on small numbers of participants with the predictor of interest. One limitation specific to studies of MOUD receipt or opioid abstinence that did not clearly establish temporality between methamphetamine/amphetamine use and the outcome of interest is the possibility that findings reflect reverse causality (i.e., the impact of receiving MOUD or using opioids during MOUD on methamphetamine/amphetamine use). However, in both outcome groups there were multiple studies that did clearly measure methamphetamine/amphetamine use prior to the outcome event that found a significant negative association. Finally, the scope of this review was limited to studies describing associations between methamphetamine/amphetamine use and MOUD-related outcomes. Future literature reviews should summarize existing research examining the impact of other specific substance use on MOUD, as well as the impact of methamphetamine/amphetamine use on treatment for other substance use disorders. Additionally, future reviews could summarize existing research examining the impact of methamphetamine/amphetamine and other substance use on sequelae of opioid use disorder among people receiving MOUD, including overdose.

### Conclusions

Methamphetamine/amphetamine use has sharply increased among people with OUD. Findings from studies identified in this systematic literature review generally suggest that methamphetamine/amphetamine use negatively impacts MOUD receipt, MOUD retention, and opioid abstinence during MOUD. Future research should examine how specific aspects of MOUD care and low-barrier models of treatment impact MOUD outcomes for this population. Research is also needed to better understand the perspectives of MOUD providers and people who use both opioids and methamphetamine/amphetamines. Continued efforts to expand and improve MOUD and overdose prevention strategies for this population are needed.

## Data Availability

Not applicable.

## Database search queries

### PubMed/MEDLINE

(amphetamine OR amphetamines OR methamphetamine OR methamphetamines OR meth OR stimulant OR stimulants OR “other drug” OR “other drugs” OR “other substance” OR “other substances” OR polydrug OR polysubstance OR “multiple drug” OR “multiple drugs” OR “multiple substance” OR “multiple substances” OR “Methamphetamine”[Mesh] OR “Amphetamine”[Mesh] OR “Amphetamine-Related Disorders”[Mesh] OR “Central Nervous System Stimulants”[Mesh]).

AND (opioid OR opioids OR opiate OR opiates OR narcotic OR narcotics OR heroin OR fentanyl OR oud OR “Opioid-Related Disorders”[Mesh] OR “Opioid Epidemic”[Mesh] OR “Heroin”[Mesh] OR “Heroin Dependence”[Mesh] OR “Opium Dependence”[Mesh] OR “Morphine Dependence”[Mesh] OR “Fentanyl”[Mesh]).

AND (treatment OR help OR pharmacotherapy OR moud OR mat OR agonist OR buprenorphine OR methadone OR naltrexone OR suboxone OR subutex OR maintenance OR substitution OR replacement OR therapy OR “Buprenorphine”[Mesh] OR “Methadone”[Mesh] OR “Naltrexone”[Mesh] OR “Opiate Substitution Treatment”[Mesh] OR “Substance Abuse Treatment Centers”[Mesh] OR “Substance-Related Disorders/rehabilitation”[Mesh] OR “Opioid-Related Disorders/rehabilitation”[Mesh] OR “Opioid-Related Disorders/therapy”[Mesh]).

AND (start OR start* OR initiat* OR engag* OR uptake OR receive OR receiv* OR receipt OR access OR access* OR enter OR enter* OR entry OR enroll OR enroll* OR admit OR admit* OR admission OR utiliz* OR retain OR retain* OR retention OR complete OR complet* OR drop OR drop* OR fail OR fail* OR discontinu* OR success OR succeed OR succeed* OR adhere OR adheren* OR comply OR complian* OR abstain OR abstain* OR abstinen* OR clean OR dirty OR urinalysis OR “urine drug test” OR “urine drug screen” OR “urine test” OR “urine screen” OR UDS OR UDT OR “Retention in Care”[Mesh] OR “Duration of Therapy”[Mesh] OR “Patient Acceptance of Healthcare”[Mesh] OR “Treatment Refusal”[Mesh] OR “Urinalysis”[Mesh]).

### PsycINFO

(amphetamine OR amphetamines OR methamphetamine OR methamphetamines OR meth OR stimulant OR stimulants OR “other drug” OR “other drugs” OR “other substance” OR “other substances” OR polydrug OR polysubstance OR “multiple drug” OR “multiple drugs” OR “multiple substance” OR “multiple substances” OR DE "Amphetamine" OR DE “Dextroamphetamine” OR DE “Methamphetamine” OR DE “Methylenedioxymethamphetamine” OR DE “CNS Stimulating Drugs” OR DE “Polydrug Abuse”).

AND (opioid OR opioids OR opiate OR opiates OR narcotic OR narcotics OR heroin OR fentanyl OR oud OR DE “Opioid Use Disorder” OR DE “Heroin Addiction” OR DE “Morphine Dependence” OR DE “Prescription Drug Misuse” DE “Heroin” OR DE “Fentanyl”).

AND (treatment OR help OR pharmacotherapy OR moud OR mat OR agonist OR buprenorphine OR methadone OR naltrexone OR suboxone OR subutex OR maintenance OR substitution OR replacement OR therapy OR DE “Addiction Treatment” OR DE “Substance Use Treatment” OR DE “Drug Therapy” OR DE “Medication-Assisted Treatment” OR DE “Maintenance Therapy” OR DE “Buprenorphine” OR DE “Naltrexone” OR DE “Methadone” OR DE “Methadone Maintenance”).

AND (start OR start* OR initiat* OR engag* OR uptake OR receive OR receiv* OR receipt OR access OR access* OR enter OR enter* OR entry OR enroll OR enroll* OR admit OR admit* OR admission OR utiliz* OR retain OR retain* OR retention OR complete OR complet* OR drop OR drop* OR fail OR fail* OR discontinu* OR success OR succeed OR succeed* OR adhere OR adheren* OR comply OR complian* OR abstain OR abstain* OR abstinen* OR clean OR dirty OR urinalysis OR “urine drug test” OR “urine drug screen” OR “urine test” OR “urine screen” OR UDS OR UDT OR DE “Drug Abstinence” OR DE “Drug Usage Screening” OR DE “Urinalysis” OR DE “Treatment Compliance” OR DE “Treatment Termination” OR DE “Treatment Duration” OR DE “Treatment Refusal” OR DE “Treatment Barriers” OR DE “Treatment Dropouts”).

### CINAHL complete

(amphetamine OR amphetamines OR methamphetamine OR methamphetamines OR meth OR stimulant OR stimulants OR “other drug” OR “other drugs” OR “other substance” OR “other substances” OR polydrug OR polysubstance OR “multiple drug” OR “multiple drugs” OR “multiple substance” OR “multiple substances” OR MH “Methamphetamine  + ” OR MH “Amphetamine  + ” OR MH “Amphetamines  + ” OR MH “Central Nervous System Stimulants  + ”).

AND [opioid OR opioids OR opiate OR opiates OR narcotic OR narcotics OR heroin OR fentanyl OR oud OR MH “Heroin  + ” OR MH “Fentanyl  + ” OR (MH “Substance Use Disorders  + ” AND MH “Analgesics, Opioid  + ”)].

AND (treatment OR help OR pharmacotherapy OR moud OR mat OR agonist OR buprenorphine OR methadone OR naltrexone OR suboxone OR subutex OR maintenance OR substitution OR replacement OR therapy OR MH “Substance Use Rehabilitation Programs  + ” OR MH “Buprenorphine  + ” OR MH “Naltrexone  + ” OR MH “Methadone  + ”).

AND (start OR start* OR initiat* OR engag* OR uptake OR receive OR receiv* OR receipt OR access OR access* OR enter OR enter* OR entry OR enroll OR enroll* OR admit OR admit* OR admission OR utiliz* OR retain OR retain* OR retention OR complete OR complet* OR drop OR drop* OR fail OR fail* OR discontinu* OR success OR succeed OR succeed* OR adhere OR adheren* OR comply OR complian* OR abstain OR abstain* OR abstinen* OR clean OR dirty OR urinalysis OR “urine drug test” OR “urine drug screen” OR “urine test” OR “urine screen” OR UDS OR UDT OR MH “Substance Abuse Detection  + ” OR MH “Urinalysis  + ” OR MH “Patient Compliance  + ” OR MH “Medication Compliance  + ” OR MH “Treatment Termination  + ” OR MH “Treatment Duration  + ” OR MH “Treatment Delay  + ” MH “Treatment Refusal  + ” OR MH “Patient Dropouts  + ”).

## Appendix 2

**Table **

**Table 5:** Detailed description of covariates in included studies

Author/pub year	Covariates included in analysis
Abrahamsson 2016	None
Banta-Green 2009	Age, gender, race/ethnicity, medical concerns, public assistance, home conducive to recovery, children under 12 at home, legal system involvement, prescription opiate use only vs. heroin use, cocaine use, treatment agency
Daniulaityte 2020	Age, gender, race, homelessness, psychiatric comorbidity, ever prescribed pharmaceutical stimulants, ever used diverted pharmaceutical stimulants, prefer fentanyl vs. heroin, injection as primary method of heroin/fentanyl administration, days of use in past 6 months of heroin/fentanyl, non-prescribed pain pills, non-prescribed buprenorphine, marijuana, cocaine, and non-prescribed benzodiazepine, lifetime receipt of other 2 types of MOUD
Deck 2004	Age, gender, race/ethnicity, Medicaid program, years of opiate use, needle use, frequency of opiate use, cocaine use, alcohol use, mental health needs (Washington only), arrested, prior methadone, prior SUD treatment, distance from clinic, referral source (self/treatment agency/legal), not employable, no source of income, marital status, housing (live in own home/live in group home/homeless/other), pregnant, months Medicaid eligible; enrolled in ADATSA (alcohol and drug abuse prevention and treatment; Washington only)
Deck 2005	Age, gender, race/ethnicity, Medicaid program, years of opiate use, needle use, frequency of opiate use, cocaine use, alcohol use, mental health needs (Washington only), arrested, prior methadone, prior SUD treatment, distance from clinic, referral source (self/treatment agency/legal), not employable, no source of income, marital status, housing (personal home/homeless/other), pregnant, state Medicaid eligibility, enrolled in ADATSA (alcohol and drug abuse prevention and treatment; Washington only), admission cohort, treatment agency
Fairbairn 2012	Age, gender, Midazolam injection, heroin injection, alcohol consumption
Gjersing 2013	None
Hall 2016	None
Hoang 2018	Time of assessment, family support, number of years used heroin prior to initiation, HIV status, antiretroviral therapy receipt
Hser 2014	Age, gender, race/ethnicity, short form 36-item health survey scores (physical component summary and mental component summary), alcohol use, number of cigarettes smoked/day, opioid-positive UDS, cannabis-positive UDS, cocaine-positive UDS, days of heroin/opiate use in past 30 days, site (west vs. east coast), dose on last day of treatment, methadone vs. buprenorphine (in total sample), interaction of buprenorphine/methadone with dose (in total sample)
Hui 2017	None
Jones 2020	Age, gender, race/ethnicity, US census region, employment status, living arrangement, treatment referral source, heroin injection, age of first heroin use
Kumar 2016	Age, gender, marital status, route of opioid use, pain, current substance use other than benzodiazepines and opioids, benzodiazepine use, cocaine use, opioid use, cannabis use, physical *or* emotional neglect (2 models)
Kunøe 2010	Age, gender
Liu 2017	Marital status, number of times in “compulsory drug detoxification”
Liu 2018	None
Lo 2018	Age, homelessness, incarceration, no income assistance, binge alcohol use, daily opioid use, daily heroin injection, daily cocaine injection, binge on drug injection, HIV, proportion of visits on methadone, methadone dose
Logan 2019	None
Manhapra 2017	None
Manhapra 2018	None
Manhapra 2020	None
Michel 2017	Frequency of injection (< 75 vs. > 75 injections/month)
Morgan 2018	*MOUD receipt (logistic regression model)*: Age, gender, geographic region, health plan type, alcohol use disorder, cannabis use disorder, cocaine use disorder, hallucinogen use disorder, sedative use disorder *MOUD retention (Cox proportional hazards model)*: Age, gender, region of residence, alcohol use disorder, cannabis use disorder, cocaine use disorder, hallucinogen use disorder, sedative use disorder, ever seen in detox facility, type of provider at initiation, place of initiation, commercial insurance type, type of MOUD, effect of medication type in first 30 days of treatment
Peles 2008	*Tel Aviv sample* : None *Las Vegas sample*: Age, have children, duration opioid use before admission, Hepatitis C
Peles 2015	None
Pettes 2010	None
Potter 2013	None
Proctor 2015	*12-month model*: Age, gender, method of payment, average daily methadone dose *6-month model* : Race/ethnicity, marital status, employment status, average daily methadone dose
Proctor 2016	*3-month model*: Age, gender, employment status, race/ethnicity, marital status, and average daily methadone dose *6-month model*: Race/ethnicity, marital status, and average daily methadone dose *9-month model*: Age, gender, employment status, race/ethnicity, marital status, and average daily methadone dose *12-month model*: Age, race/ethnicity, employment status, and average daily methadone dose
Rhee 2019	None
Schiff 2007	Age, gender, age × gender interaction, heroin use, cocaine use, benzodiazepine use, cannabis use
Schuman-Olivier 2014	None
Senbanjo 2009	None
Shiner 2017	Age, gender, race/ethnicity, marital status, rural vs. urban, VA disability level, homelessness, OEF/OIF/OND veteran, combat exposure, sexual trauma while in military, Charlson comorbidity index, plurality of care at VA medical center, plurality of care at a CBOC, ≥ 1 visit with a primary care prescriber, ≥ 1 visit with a mental health prescriber, ≥ 1 visit with a SUD prescriber, residential SUD treatment, inpatient admission for detoxification, effective antidepressant for PTSD, pain disorder, headache disorder, psychotic disorders, bipolar mood disorders, depressive mood disorders, non-PTSD anxiety disorders, TBI and cognitive disorders, personality disorders, nicotine use disorder, alcohol use disorder, marijuana use disorder, cocaine use disorder, amphetamine use disorder, tranquilizer and sedative use disorder, hallucinogen use disorder, fiscal year
Skeie 2013	None
Smyth 2018	None
Thirion 2001	None
Tsui 2020	Age, gender, race/ethnicity, education level, clinic site, time period of enrollment
White 2014	None

**5**

## Abbreviations

FDA: US Food and Drug Administration
MOUD: Medications for opioid use disorder
OUD: Opioid use disorder
PRISMA: Preferred reporting items for systematic reviews and meta-analysis
PWID: People who inject drugs
UDS: Urine drug screen
